# Increased myocardial mass and attenuation of myocardial strain in professional male soccer players and competitive male triathletes

**DOI:** 10.1007/s10554-020-01918-1

**Published:** 2020-06-20

**Authors:** Jitka Starekova, Tilo Thottakara, Gunnar K. Lund, Götz H. Welsch, Fabian J. Brunner, Kai Muellerleile, Gerhard Adam, Marc Regier, Enver Tahir

**Affiliations:** 1 Department of Diagnostic and Interventional Radiology and Nuclear Medicine, University Medical Center Hamburg-Eppendorf, Martinistr. 52, 202 46 Hamburg, Germany; 2 Department of Cardiology, University Heart and Vascular Center, Martinistr. 52, 20246 Hamburg, Germany; 3 Center for Athletic Medicine - Athleticum, University Medical Center Hamburg-Eppendorf, Martinistr. 52, 202 46 Hamburg, Germany

**Keywords:** Athlete´s heart, Cardiac hypertrophy, Magnetic resonance imaging, CMR feature tracking, Myocardial strain

## Abstract

The purpose of this prospective study was to analyze the relationship between ventricular morphology and parameters of cardiac function in two different athletic groups and controls, using feature tracking cardiac magnetic resonance (FT-CMR). Twenty-three professional soccer players (22 ± 4 years), 19 competitive triathletes (28 ± 6 years) and 16 controls (26 ± 3 years) were included in the study. CMR was performed using a 1.5 T scanner. Cardiac chamber volumes, mass and biventricular global myocardial strain were obtained and compared. In comparison to the control subjects, athletes were characterized by a higher cardiac volume (*p* < 0.0001), higher cardiac mass (*p* < 0.001), reduced longitudinal strain of the left and right ventricle (*p* < 0.05 and *p* < 0.01 respectively) and reduced left ventricular radial strain (*p* < 0.05). Soccer players revealed higher amounts of left ventricular mass (87 ± 15 vs. 75 ± 13 g/m^2^, *p* < 0.05) than triathletes. Moreover, they showed a greater decrease in left and right ventricular longitudinal strain (*p* < 0.05 and *p* < 0.05) as well as in radial left ventricular strain (*p* < 0.05) in comparison to triathletes. An increase in left ventricular mass correlated significantly with a decrease in longitudinal (*r* = 0.47, *p* < 0.001) and radial (*r* =  − 0.28, *p* < 0.05) strain. In athletes, attenuation of strain values is associated with cardiac hypertrophy and differ between soccer players and triathletes. Further studies are needed to investigate whether it is an adaptive or maladaptive change of the heart induced by intense athletic training.

## Introduction

Morphological changes of the heart associated with exercise are well studied by both echocardiography and cardiac magnetic resonance (CMR) imaging [1–5]. However, changes in myocardial mechanics of athlete’s heart are less understood. While some echocardiographic studies reported no change in left ventricular (LV) strain in endurance athletes, others observed a reduction of apical radial strain [6–8]. Myocardial strain describes deformation (relative change of the fiber length) of the myocardium during the cardiac cycle and is a marker of systolic function [9, 10]. Feature tracking CMR (FT-CMR) is an imaging technique that allows quantification of myocardial strain as well as strain rates using short and long-axis cine images [9, 11]. The level of cardiac hypertrophy and dilatation of the heart chambers may vary depending on the type and extent of the sport activity [1–5]. To understand the changes of myocardial strain in correlation with the degree of exercise-induced myocardial hypertrophy is important. Excessive myocardial hypertrophy due to a high level of athletic activity was previously linked to focal myocardial fibrosis and higher blood pressure response during exercise. However, left ventricular ejection fraction (LVEF) of athletes with focal myocardial fibrosis was preserved [12]. Myocardial strain is an early marker of systolic dysfunction, which precedes a decline in LVEF [9] and might therefore be an important parameter when evaluating athlete´s heart. We hypothesized that myocardial deformation might be influenced by the different types of sports activity. The purpose of this prospective study was to assess ventricular morphology and parameters of systolic function between two athletic groups, professional soccer players and amateur triathletes using FT-CMR.

## Materials and methods

### Subjects

The ethics committee of the general medical council approved the study. The subjects were recruited between 2014 and 2017 and gave their written informed consent. Twenty-three male professional soccer players (22 ± 4 years, range 18–32 years) were recruited during their competitive season in the center for athletic medicine. Nineteen male amateur triathletes (28 ± 6 years, range 18–36 years) were contacted through advertisement at local triathlon clubs and were included if they had participated in at least 10 h of weekly training and regular participation in triathlon competitions in the past 3 years. Sixteen male control subjects (26 ± 3 years, range 20–31 years) with < 3 h exercise per week were recruited through local advertisement. Study exclusion criteria were contraindications for CMR or any disease. All subjects were instructed to arrive rested with no exercise in the preceding 72 h. Food and caffeine intake were restricted in the preceding 3 h.

### CMR protocol

Non-contrast CMR was performed using a 1.5T Achieva scanner equipped with a 5-channel cardiac phased array receiver coil (Philips Healthcare, Best, The Netherlands). The protocol included ECG-triggered steady-state free-precession cine CMR in short axis and 2-, 3- and 4-chamber views with the following imaging parameters: acquired voxel size (AVS) 1.98 × 1.80 × 6 mm^3^, reconstructed voxel size (RVS) 1.36 × 1.36 × 6 mm^3^, gap 4 mm, 9–10 slices for full LV coverage, echo time = 1.67 ms, repetition time = 3.34 ms, flip angle = 60°, parallel acquisition technique = SENSE.

### CMR data analysis

Two investigators independently and blindly analyzed each CMR using cvi42 software (Circle Cardiovascular Imaging Inc, Calgary, Alberta, Canada). CMR parameters were indexed to body surface area (BSA) and are given as the mean of two observers. Evaluation of ventricular volumes and LV mass was performed in standard fashion on short axis cine images [13]. Left (LA) and right atrial (RA) volumes and ejection fraction (EF) were quantified using the biplane area-length method, excluding pulmonary veins and atrial appendage [14]. The interventricular septum (IVS) thickness and lateral wall thickness of LV in end-diastole were measured on a basal short axis slice immediately basal to the tips of the papillary muscles in end-diastole [13]. Relative wall mass ratio was calculated as a ratio of LV mass index/LV end-diastolic volume index (LVEDVi) [15]. Septal/lateral wall thickness ratio was calculated as a ratio of wall thickness of septal and lateral segments [15]. LV cavity diameter was measured in 3-chamber view at the mitral chordae level basal to the tips of papillary muscles [13]. Peak systolic LV and right ventricular (RV) strain as well as diastolic strain rate were analyzed with cine CMR images using Segment feature tracking software version 2.1.R.6108 (Medviso, Lund, Sweden). This software analyzes myocardial strain and diastolic strain rate by computing interframe deformation fields using an endocardial tracking strategy based on non-rigid image registration [9, 16]. LV global peak systolic radial, longitudinal and circumferential strain were measured on three long and three short axis cine slices by manual delineation of the endo- and epicardial contours in end-diastole. RV global peak systolic longitudinal and circumferential strain and diastolic strain rate were measured on a single 4-chamber and three short axis cine slices by manual delineation of the endocardial contours in end-diastole. Contours were then automatically propagated by the software throughout the cardiac cycle generating myocardial strain [9]. Diastolic function was assessed with a dedicated software (CMRtools^®^, Cambs, UK) [17]. Briefly, LV volumetry was performed using manual delineation of the LV endocardial borders in end-diastolic, end-systolic and mid-diastolic short axis views, trabeculae and papillary muscles were excluded from the LV cavity and time-volume-curves from all time frames of the cardiac cycle were calculated [17, 18]. The differentiated time-volume-curve is characterized by two diastolic peaks including the early peak-filling rate (EPFR) and atrial peak-filling rate (APFR), which represent the maximum speed of passive LV filling and the maximum speed of LV filling secondary to atrial contraction. The peak filling rate ratio (PFRR) describes the contribution of each in diastolic LV filling and is calculated as EPFR/APFR [17, 19].

### Statistical analysis

Statistical analysis was performed using GraphPad Prism version 6.00 (GraphPad Software, San Diego, CA, USA) and MedCalc for Windows, version 13.3.3.0 (MedCalc Software, Ostend, Belgium). Continuous data are presented as mean ± standard deviation (SD) and categorical data are presented as absolute numbers and percentages. Continuous data were compared using the two-sided Student's t-test and categorical variables were compared using the *χ*^2^ test or Fischer’s exact test as appropriate. Statistical significance was defined as *p* < 0.05.

## Results

### Demographics of athletes and controls

There were no significant differences in age, weight, height and in BSA between athletes and controls (Table 1). However, athletes had a lower body mass index (BMI) compared to controls (*p* < 0.05). The soccer players were younger than the triathletes (*p* < 0.05), but weight, height, BMI and BSA did not differ (Table 2).

**Table 1:** Demographics and CMR parameters of athletes (soccer players and triathletes) and sedentary controls

	Athletes (*n* = 42)	Controls (*n* = 16)	*p* value
Demographics			
Age (years)	25 ± 5	26 ± 3	0.291
Weight (kg)	76 ± 9	80 ± 14	0.173
Height (m)	1.83 ± 0.06	1.82 ± 0.08	0.437
BMI (kg/m^2^)	23 ± 2	24 ± 3	**< 0.05**
BSA (m^2^)	1.97 ± 0.13	2.00 ± 0.21	0.495
CMR left heart			
LV ejection fraction (%)	58 ± 3	59 ± 3	0.239
IVS thickness in ED (mm)	11.1 ± 1.5	9.4 ± 1.0	**< 0.001**
LV lateral wall thickness in ED (mm)	10.2 ± 1.6	8.7 ± 1.0	**< 0.01**
Septal/lateral wall thickness ratio	1.1 ± 0.2	1.1 ± 0.2	0.643
LV diameter in ED (mm)	5.6 ± 0.3	5.4 ± 0.4	**< 0.05**
LVMi (g/m^2^)	82 ± 15	67 ± 7	** < 0.001**
LVEDVi (ml/m^2^)	105 ± 11	91 ± 10	**< 0.0001**
LVESVi (ml/m^2^)	44 ± 5	37 ± 6	**< 0.0001**
LVSVi (ml/m^2^)	61 ± 8	54 ± 6	**< 0.01**
Relative wall mass ratio (LVMi/LVEDVi)	0.78 ± 0.11	0.74 ± 0.08	0.195
LAEDVi (ml/m^2^)	19 ± 6	14 ± 5	**< 0.01**
CMR right heart			
RV ejection fraction (%)	57 ± 5	59 ± 5	0.503
RVEDVi (ml/m^2^)	103 ± 13	92 ± 12	**< 0.01**
RVESVi (ml/m^2^)	43 ± 8	38 ± 8	**< 0.05**
RVSVi (ml/m^2^)	60 ± 9	54 ± 7	**< 0.05**
RAEDVi (ml/m^2^)	26 ± 8	19 ± 6	**< 0.001**
Strain parameters			
LV radial (%)	40 ± 8	45 ± 9	**< 0.05**
LV longitudinal (%)	− 16 ± 2	− 18 ± 1	**< 0.05**
LV circumferential (%)	− 15 ± 3	− 16 ± 3	0.141
RV longitudinal (%)	− 19 ± 3	− 22 ± 2	**< 0.01**
RV circumferential (%)	− 9 ± 3	− 9 ± 4	0.899

Values are mean ± SD for continuous data. Bold characters indicate statistically significant values (*p* < 0.05)

*BMI* body mass index, *BSA* body surface area, *ED* end-diastole, *IVS* interventricular septum, *LAEDVi* left atrial end-diastolic volume index, *LVEDVi* left ventricular end-diastolic volume index, *LV* left ventricular, *LVESVi* left ventricular end-systolic volume index, *LVMi* left ventricular mass index, *LVSVi* left ventricular stroke volume index, *RAEDVi* right atrial end-diastolic volume index, *RV* right ventricular, *RVEF* right ventricular ejection fraction, *RVEDVi* right ventricular end-diastolic volume index, *RVESVi* right ventricular end-systolic volume index, *RVSVi* right ventricular stroke volume index

**Table 2:** Demographics and CMR parameters of professional soccer players and competitive triathletes

	Soccer players (*n* = 23)	Triathletes (*n* = 19)	*p* value
Demographics			
Age (years)	22 ± 4	28 ± 6	**< 0.001**
Weight (kg)	77 ± 9	74 ± 8	0.290
Height (m)	1.83 ± 0.07	1.83 ± 0.06	0.855
BMI (kg/m^2^)	23 ± 2	22 ± 2	0.116
BSA (m^2^)	1.99 ± 0.14	1.96 ± 0.13	0.491
CMR—left heart			
LV ejection fraction (%)	58 ± 4	59 ± 3	0.328
Heart rate (beats/min)	53 ± 9	56 ± 10	0.391
IVS thickness in ED (mm)	11.5 ± 1.7	10.5 ± 1.1	**< 0.05**
LV lateral wall thickness in ED (mm)	10.9 ± 1.5	9.3 ± 1.4	**< 0.01**
Septal/lateral wall thickness ratio	1.07 ± 0.2	1.13 ± 0.1	0.183
LV diameter in ED (mm)	5.6 ± 0.3	5.6 ± 0.3	0.890
LVMi (g/m^2^)	87 ± 15	75 ± 13	**< 0.05**
LVEDVi (ml/m^2^)	107 ± 10	102 ± 12	0.168
LVESVi (ml/m^2^)	45 ± 5	42 ± 5	0.071
LVSVi (ml/m^2^)	62 ± 8	60 ± 8	0.467
Relative wall mass ratio (LVMi/LVEDVi)	0.82 ± 0.12	0.74 ± 0.10	**< 0.05**
LAEDVi (ml/m^2^)	20 ± 6	17 ± 5	0.272
CMR—right heart			
RV ejection fraction (%)	57 ± 5	59 ± 5	0.222
RVEDVi (ml/m^2^)	104 ± 13	102 ± 14	0.510
RVESVi (ml/m^2^)	45 ± 8	42 ± 9	0.242
RVSVi (ml/m^22^)	60 ± 9	60 ± 9	0.991
RAEDVi (ml/m^2^)	28 ± 8	23 ± 7	**< 0.05**
Strain parameters			
LV radial (%)	38 ± 8	43 ± 7	**< 0.05**
LV longitudinal (%)	− 16 ± 2	− 17 ± 2	**< 0.05**
LV circumferential (%)	− 15 ± 3	− 15 ± 2	0.634
RV longitudinal (%)	− 18 ± 2	− 20 ± 3	**< 0.05**
RV circumferential (%)	− 9 ± 3	− 8 ± 2	0.101

Values are mean ± SD for continuous data. Bold characters indicate statistically significant values (*p* < 0.05)

*BMI* body mass index, *BSA* body surface area, *ED* end-diastole, *IVS* interventricular septum, *LAEDVi* left atrial end-diastolic volume index, *LVEDVi* left ventricular end-diastolic volume index, *LV* left ventricular, *LVESVi* left ventricular end-systolic volume index, *LVMi* left ventricular mass index, *LVSVi* left ventricular stroke volume index, *RAEDVi* right atrial end-diastolic volume index, *RV* right ventricular, *RVEF* right ventricular ejection fraction, *RVEDVi* right ventricular end-diastolic volume index, *RVESVi* right ventricular end-systolic volume index, *RVSVi* right ventricular stroke volume index

### Cardiac function, volumes and mass assessed by CMR

While IVS thickness in athletes was increased compared to controls (11.1 ± 1.5 mm vs. 9.4 ± 1 mm, *p* < 0.001) septal/lateral wall thickness ratio did not differ between groups (*p* = 0.643 Table 1). The LV mass index was markedly higher in athletes with 82 ± 15 g/m^2^ than in controls with 67 ± 7 g/m^2^ (*p* < 0.001). The indexed end-diastolic LV volume (LVEDVi) was significantly higher in athletes than in controls (105 ± 11 vs. 91 ± 10 ml/m^2^, *p* < 0.0001), which was also true for the indexed end-diastolic RV volume (103 ± 13 vs. 92 ± 12 ml/m^2^, *p* < 0.01, Table 1). The relative wall mass ratio did not differ between the athletes and controls (*p* = 0.195, Table 1).

Soccer players had a higher LV mass index with 87 ± 15 g/m^2^ than triathletes with 75 ± 13 g/m^2^ (*p* < 0.05, Table 2). Also, they had a higher relative wall mass ratio compared to triathletes (0.82 ± 0.12, 0.74 ± 0.10, *p* < 0.05). Furthermore, IVS thickness of soccer players was higher compared to triathletes (*p* = 11.5 ± 1.7 vs. 10.5 ± 1.1 mm, *p* < 0.05, Table 2). Septal/lateral wall thickness ratio did not differ between the groups (*p* = 0.183, Table 2). Indexed end-diastolic LV and RV volumes did not differ between the athletic groups (*p* = 0.168 and p = 0.510, Table 2).

### Strain and strain rate analysis by feature tracking CMR

LV global radial and longitudinal strain were lower in athletes with 40 ± 8% and − 16 ± 2%, compared to the sedentary controls with 45 ± 9% (*p* < 0.05) and − 18 ± 1% (*p* < 0.05) respectively (Table 1). Similarly, RV global longitudinal strain was lower in athletes with − 19 ± 3% compared to the controls with − 22 ± 2% (*p* < 0.01). Global circumferential LV (*p* = 0.141) and RV (*p* = 0.899) strain values were similar between groups (Table 1). LV global radial and longitudinal strain were lower in soccer players with 38 ± 8% and − 16 ± 2% compared to triathletes with 43 ± 7% (*p* < 0.05) and − 17 ± 2% (*p* < 0.05), respectively. Soccer players had a lower RV global longitudinal strain with − 18 ± 2% than triathletes with − 20 ± 3% (*p* < 0.05, Table 2). Global circumferential LV (*p* = 0.634) and RV (*p* = 0.101) strain values were similar between groups (Table 2).

LV (59 ± 15 vs. 71 ± 17%/s, *p* < 0.01) and RV (74 ± 25 vs. 100 ± 25%/s, *p* < 0.01) diastolic longitudinal strain rates were lower in athletes compared to controls. LV and RV diastolic circumferential strain rates showed a tendency for lower values in athletes compared to controls (Table 3). LV diastolic radial strain rate (*p* = 0.221) did not differ. LV diastolic longitudinal strain rate (54 ± 11 vs. 64 ± 18%/s, *p* < 0.05) was lower in soccer players compared to triathletes. Concordantly, RV diastolic longitudinal strain rate (*p* = 0.065) showed a tendency for lower values in soccer players, but other diastolic strain rates values did not differ (Table 4).

**Table 3:** CMR-Derived diastolic function in athletes (soccer players and triathletes) and sedentary controls

Diastolic function	Athletes (*n* = 42)	Controls (*n* = 16)	*p* value
EPFRi (ml/s/m^2^)	242 ± 71	234 ± 68	0.759
APFRi (ml/s/m^2^)	101 ± 44	165 ± 72	**0.0001**
PFRR (EPFRi/APFRi)	2.6 ± 1.2	1.6 ± 0.6	**< 0.01**
LV diastolic radial strain rate (%/s)	− 214 ± 62	− 237 ± 64	0.221
LV diastolic longitudinal strain rate (%/s)	59 ± 15	71 ± 17	**< 0.01**
LV diastolic circumferential strain rate (%/s)	67 ± 16	76 ± 22	0.088
RV diastolic longitudinal strain rate (%/s)	74 ± 25	100 ± 25	**< 0.01**
RV diastolic circumferential strain rate (%/s)	41 ± 12	49 ± 22	0.073

Values are mean ± SD for continuous data. Bold characters indicate statistically significant values (*p* < 0.05)

*LV* left ventricular, *RV* right ventricular, *EPFRi* early peak filling rate index, *APFRi* atrial peak filling rate index, *PFRR* peak filling rate ratio

**Table 4:** CMR-derived diastolic function in soccer players and triathletes

Diastolic function	Soccer players (*n* = 23)	Triathletes (*n* = 19)	*p* value
EPFRi (ml/s/m^2^)	242 ± 71	237 ± 83	0.835
APFRi (ml/s/m^2^)	101 ± 44	105 ± 41	0.756
PFRR (EPFRi/APFRi)	2.8 ± 1.3	2.5 ± 1.1	0.504
LV diastolic radial strain rate (%/s)	− 206 ± 60	− 224 ± 65	0.364
LV diastolic longitudinal strain rate (%/s)	54 ± 11	64 ± 18	**< 0.05**
LV diastolic circumferential strain rate (%/s)	70 ± 15	63 ± 17	0.158
RV diastolic longitudinal strain rate (%/s)	67 ± 22	82 ± 27	0.065
RV diastolic circumferential strain rate (%/s)	39 ± 11	43 ± 13	0.338

Values are mean ± SD for continuous data. Bold characters indicate statistically significant values (*p* < 0.05)

*LV* left ventricular, *RV* right ventricular, *EPFRi* early peak filling rate index, *APFRi* atrial peak filling rate index, *PFRR* peak filling rate ratio

### LV diastolic function assessed by CMR

Passive LV filling was similar between athletes and controls with comparable EPFRi values (*p* = 0.759, Table 3). However, APFRi was lower with 101 ± 44 ml/s/m^2^ in athletes than in sedentary controls 165 ± 72 ml/s/m^2^ (*p* = 0.0001) leading to higher PFRR in athletes compared to the controls (2.6 ± 1.2 vs. 1.6 ± 0.6, *p* < 0.01). No differences in EPFRi (*p* = 0.835), APFRi (*p* = 0.756) and PFRR (*p* = 0.504) were found between soccer players and triathletes as shown in Table 4.

### Correlation of LV morphology and systolic function with strain parameters

**Fig. 1:**
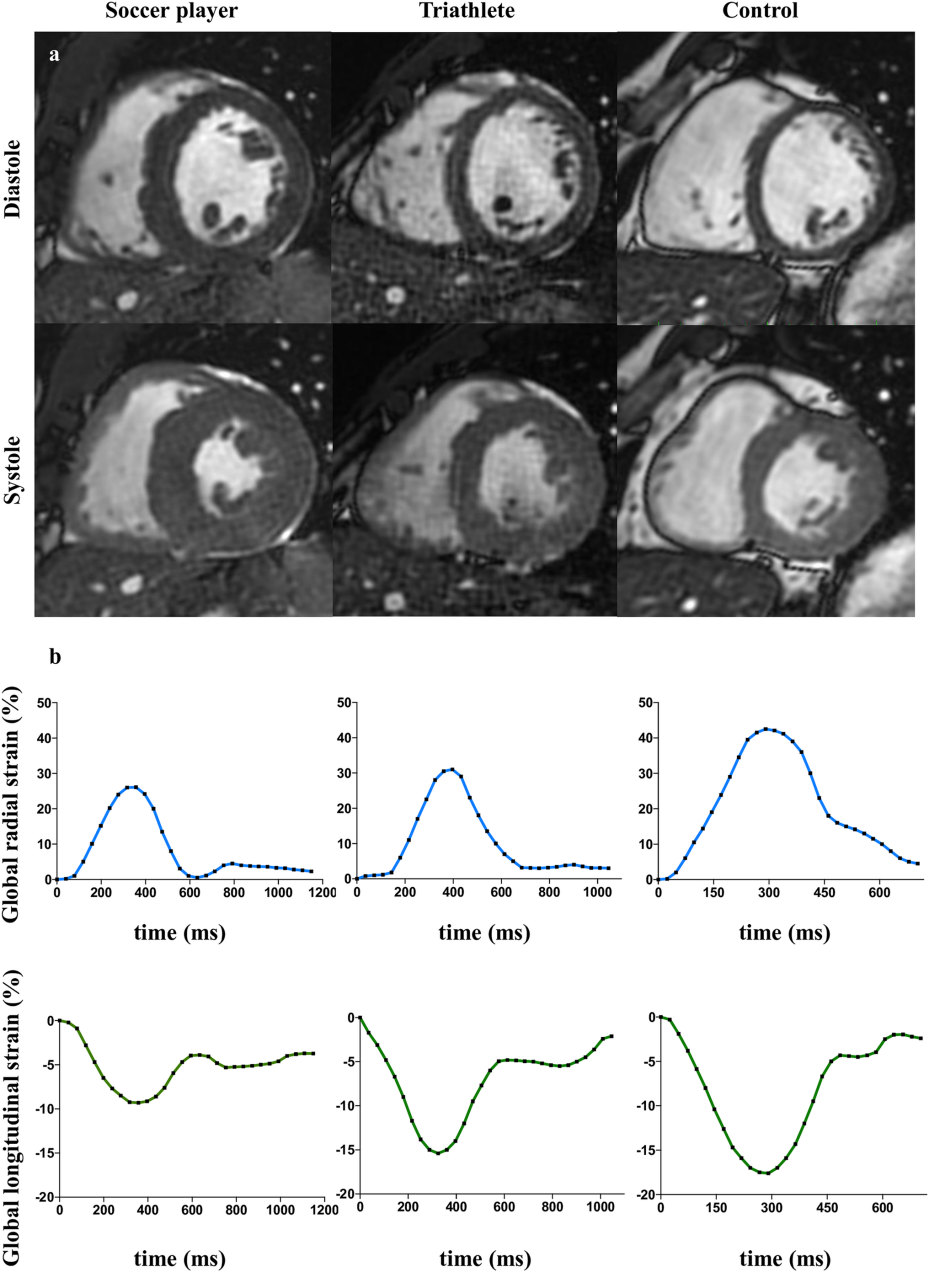
Left ventricular mass and myocardial strain. Representative short axis cine CMR images at papillary muscle level in systole and diastole (**a**). In comparison to controls, both athlete groups were characterized by higher myocardial mass. This was pronounced in professional soccer players. Increase in left ventricular mass correlated with a decrease in radial and longitudinal strain (**b**)

**Fig. 2:**
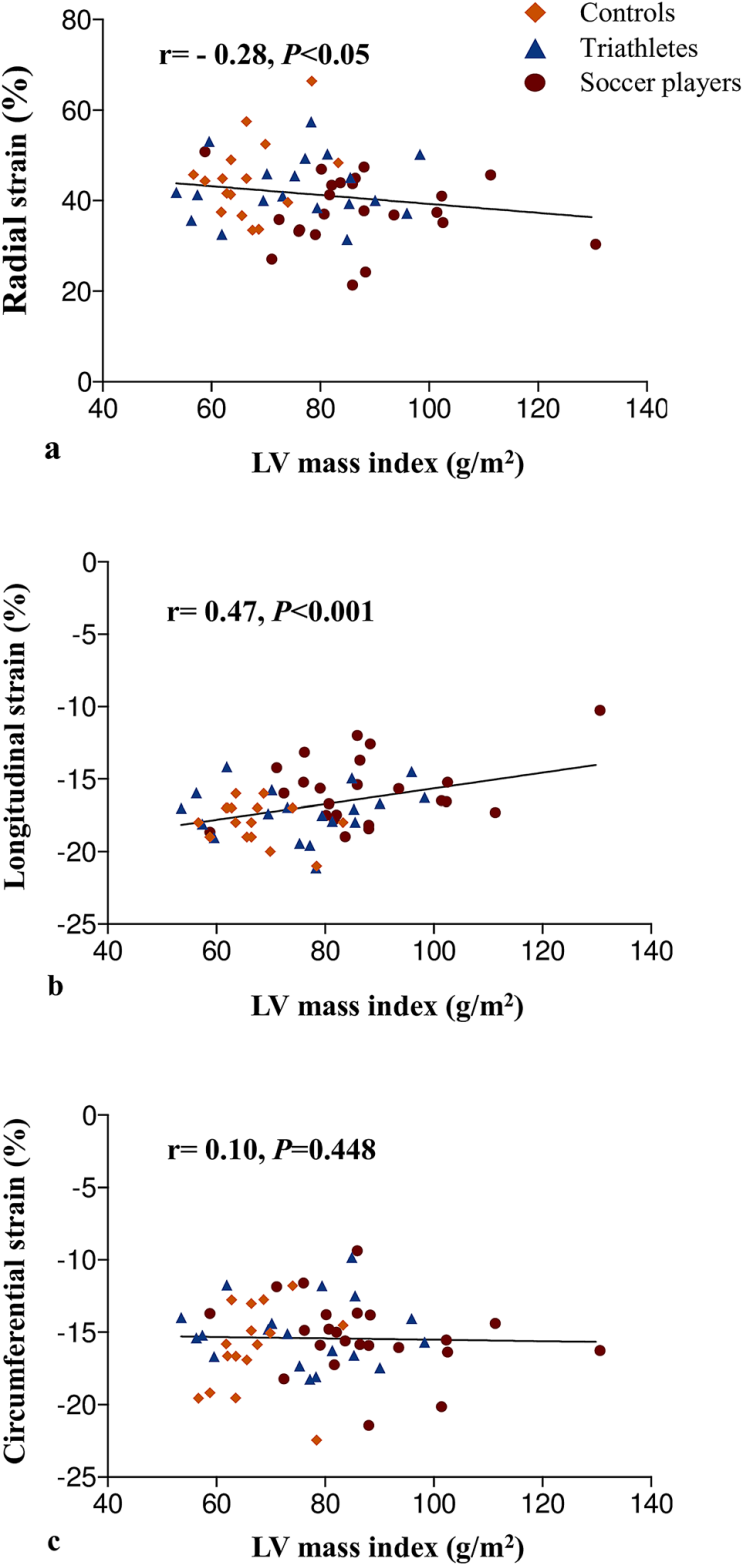
Correlations between left ventricular mass index and strain parameters. Increase in left ventricular mass index was associated with decrease in radial (**a**) and longitudinal strain (**b**), but not circumferential (**c**) strain

**Fig. 3:**
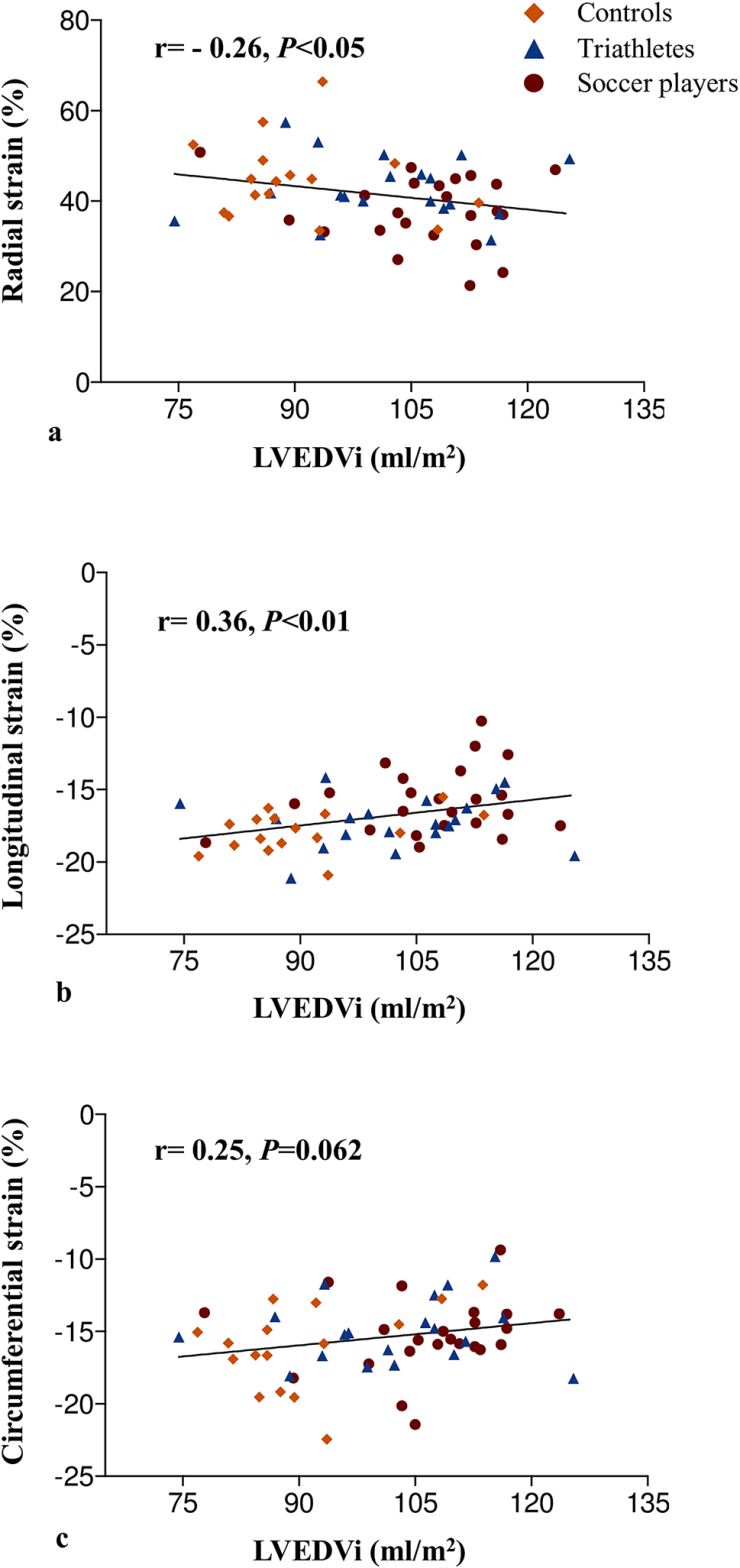
Correlations between indexed end-diastolic left ventricular volume and strain parameters. An increase in left ventricular volume was associated with a decrease in radial (**a**), longitudinal (**b**), but not significantly with circumferential (**c**) strain

**Fig. 4:**
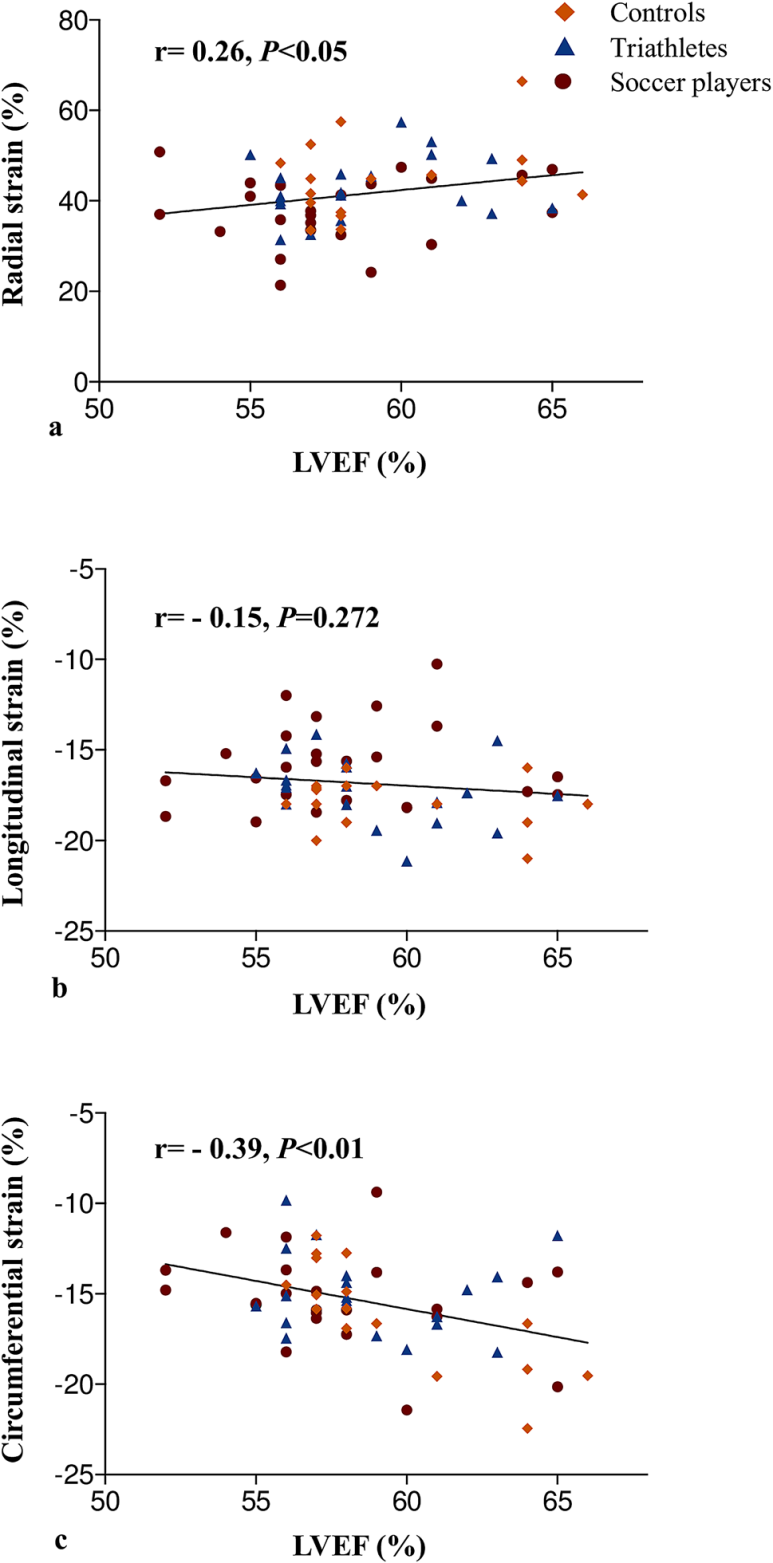
Correlations between left ventricular ejection fraction and strain parameters. An increase in left ventricular ejection fraction was associated with an increase in radial (**a**) and circumferential (**c**) strain, but not longitudinal (**b**) strain

An increase in LV mass index (Fig. 1) correlated with a decrease in radial (*r* =  − 0.28, *p* < 0.05, Fig. 2a) and longitudinal strain (*r* = 0.47, *p* < 0.001, Fig. 2b), but not with circumferential strain (*r* = 0.10, *p* = 0.448; Fig. 2c). An increase of LVEDVi was associated with a decrease in radial (*r* =  − 0.26, *p* < 0.05; Fig. 3a) and longitudinal strain (*r* = 0.36, *p* < 0.01; Fig. 3b), but not with circumferential strain (*r* = 0.25, *p* = 0.062; Fig. 3c). LVEF was associated with radial (*r* = 0.26, *p* < 0.05) and circumferential strain (*r* =  − 0.39, *p* < 0.01), but not with longitudinal strain (*r* =  − 0.15, *p* = 0.272; Fig. 4a–c).

## Discussion

This study analyzed parameters of systolic and diastolic function in correlation with exercise-induced changes in cardiac morphology, between professional soccer players and triathletes in comparison to sedentary controls using CMR. The major findings were: (1) athletes had significantly reduced global radial and longitudinal strain compared to controls, while global circumferential strain did not differ, (2) professional soccer players had significantly higher LV mass and reduced biventricular global myocardial strain values in comparison to triathletes, (3) an increase in LV mass correlated with a decrease in radial and longitudinal strain.

In line with findings of previous studies, we observed larger ventricular dimensions in professional soccer players and competitive triathletes than in sedentary controls [4, 20]. Both athlete groups were characterized by eccentric hypertrophy [15], which was pronounced in soccer players. Scharf et al. reported on professional soccer players and triathletes in two separate studies using CMR and observed larger ventricular cavities, an increase of the myocardial mass and an unchanged LVEF in comparison to controls [2, 3]. Interestingly, a direct comparison of BSA-matched soccer players and triathletes in our study showed a significantly higher LV mass in the former, while parameters of ventricular dimension did not differ. Furthermore, soccer players were characterized by higher relative wall mass ratio and greater LV wall thickness in end-diastole. One explanation for this observation might be the different type of exercise. While triathlon primarily consists of high dynamic training, soccer requires high dynamic, but also strength training [21, 22]. Thus, one can assume that the strength component of training might be a contributor to the greater LV hypertrophy in soccer players. So far characterization of myocardial strain in athletes was primarily done by echocardiography. No differences in LV strain were observed between power athletes and top-level rowers compared to controls [7, 23]. However, cyclists and professional soccer players have been characterized by decreased LV apical radial and longitudinal strain [6, 24]. Swoboda et al. reported lower LV circumferential strain in endurance athletes as assessed with CMR tissue tagging, whereas peak LV longitudinal strain by FT-CMR was similar to controls [25]. Why different athletic activities have varying contribution to attenuation of myocardial strain remains elusive. Our findings support the concept that LV hypertrophy and ventricular enlargement intrinsic to athletes including top-level soccer players are the main contributors to the decrease in biventricular strain parameters [22]. Accordingly, we found a significant negative correlation of radial and longitudinal LV strain with LV mass and end-diastolic volume, but no correlations with circumferential strain. Longitudinal strain is believed to primarily represent the contraction of the subendocardial fibers, whereas contraction of the subepicardial fibers contributes to circumferential shortening, and both aspects contribute to radial thickening [26]. Subclinical myocardial dysfunction caused by hypertension is reported to cause pathology at the subendocardial level affecting longitudinal strain first and inducing a compensatory increase of circumferential strain through hypertrophy of the subepicardial fibers [27]. Soccer players in our study showed lower longitudinal and radial strain values, preserved circumferential strain and higher level of LV hypertrophy in comparison to both triathletes and controls. Thus, it can be postulated that chronic pressure and volume overload, intrinsic to extreme athletic activity, might lead to a gradual change of the subendocardial and subepicardial myocardial fibers characterized by attenuation of longitudinal and radial strain and sustained circumferential strain to guarantee preserved LVEF. This notion could be supported by the positive correlation of LV circumferential strain with LVEF in our study, which is also in line with previous studies [28]. Hinojar et al. studied 74 HCM patients by FT-CMR. All patients showed attenuation of all strain values of the LV. Furthermore, this study demonstrated that the degree of LV hypertrophy and the amount of LV fibrosis were independent predictors of the impairment of LV mechanics, especially LV mass seemed to be the most important factor affecting myocardial strain [29]. Presence of myocardial fibrosis in athletes has been reported to occur in up to 50% of asymptomatic athletes [12, 30]. Therefore, these changes might also represent true impairment in LV strain. However, attenuation of strain values as an adaptive change is possible. Lower EF of athletes at rest as a result of a higher chamber volumes is overall accepted as an adaptive effect demonstrating increased effectiveness of athletes heart [31]. Similarly, less myocardial deformation might be needed to eject a similar stroke volume. In an echocardiographic study, high level athletes with higher chamber- and stroke volumes showed lower longitudinal strain values in comparison to low level athletes [32]. In our study athletes had similar chamber volumes, while differing in myocardial mass and strain. This suggests that myocardial mass rather than ventricular volume determines strain reduction. Notably, in pathological hypertrophy (HCM or Hypertensive heart disease) reduced strain is associated with a normal or increased EF [23, 29]. In fact, patients with hypertension were reported to have lower global longitudinal strain values in comparison to athletes [7]. In our population, the changes in strain were moderate and differentiating athletes from non-athletes might deem difficult. To fully understand the impact of exercise induced cardiac hypertrophy associated with strain attenuation in athletes, further studies are needed.

Analysis of diastolic parameters showed significant increase of PFRR in athletes compared to controls. This was driven primarily by lower APFR, which is in line with previously described decrease in A-wave peak velocity in echocardiographic studies [33–35]. EPFR did not differ between groups, confirming echocardiographic data on constant E-wave peak velocities [33–35]. In an echocardiographic study by Santoro et al. a significantly higher E/A ratio in endurance athletes was observed, caused by a decrease of the late (atrial) peak diastolic filling velocity [33]. It seems that endurance training leads to increased relaxation and elasticity of the LV at the end of diastole with secondary effects on peak atrial diastolic filling [36]. While increased PFRR suggests improved diastolic function, diastolic strain rates were decreased in our athletes.

Some limitations need to be addressed. Currently, no longitudinal data is available to study the prognostic implications of the observed decreased LV longitudinal and radial strain parameters in subjects involved in professional or competitive sports. We cannot exclude presence of the myocardial fibrosis in our study cohort and its potential influence on LV strain parameters. Our cohort included male subjects only. Therefore, our findings can only be extended to healthy male athletes [37]. The numeric strain values might vary using a different software [38]. Further, evaluation of diastolic function in echocardiography comprises of many parameters and using CMR only evaluation of EPFR, APFR, and PFRR is feasible. Additional echocardiographic parameters such as *E*/*e*′, measurement of tricuspid regurgitation velocity, *e*′ velocity and pulmonary vein doppler velocity is missing and would be required to fully understand changes of diastolic function in CMR [39]. Furthermore, LA volume as an important parameter of diastolic function is only of limited use since all athletes have enlarged heart chambers. This study is also limited by the small number of subjects.

In conclusion, our study revealed that attenuation of longitudinal and radial strain values is associated with the level of sport induced myocardial hypertrophy in athletes and differ between soccer players and triathletes. Further studies are needed to investigate the impact of increased hypertrophy in athletes on cardiac function.
